# Environmental Protection or Development? Multiple Policy Effects Evaluation of the Resource Tax Collection Reform for Iron Ore Enterprises in China

**DOI:** 10.3390/ijerph20053976

**Published:** 2023-02-23

**Authors:** Yanhui Yin, Bowen Yang

**Affiliations:** 1 College of Economics and Trade, Shandong Management University, Jinan 250357, China; 2 College of Humanities and Social Development, Nanjing Agricultural University, Nanjing 210095, China

**Keywords:** iron ore resource tax, environmental governance, green economy recovery, green tax system

## Abstract

The change from quantity-based taxation to price-based taxation of iron ore resources is an important measure for China to implement the goal of carbon peaking and carbon neutralization, and to achieve green economic recovery. To explore the policy’s effectiveness in playing its tax function, and improving the environment and production efficiency, this paper takes the reform of the method of resource tax collection as the “quasi natural experiment” object, and selects the balanced panel data of 16 provinces in China from 2011 to 2021. The double difference method is used to evaluate the policy effect of the reform of resource tax collection. The research shows that: (1) Changing the resource tax from a “volume-based tax” to an “ad valorem tax” can effectively increase the government’s resource tax revenue, and promote the upgrading of enterprise production technology. (2) The reform of resource tax collection will eliminate some small and medium-sized enterprises that are backward in production technology and bring more pollution to the environment. (3) The reform of resource tax collection mode will increase the number of large and medium-sized iron ore enterprises and promote the standardization of the whole iron ore industry.

## 1. Introduction

The reform of the iron ore resource tax is an important measure for China to comprehensively deepen the reform of the fiscal and tax system, and is the concrete implementation of the development strategy of energy conservation and environmental protection [1]. The iron ore resource tax will be levied on a volume basis, which will lead to the disconnection between the tax and the price of iron ore resource products, and will not play its due role in the structural adjustment, technological upgrading, and innovation incentives of the whole industry. Therefore, the reform of the iron ore resource tax is very important for environmental protection and economic development.

China’s iron ore resource tax is not conducive to the protection of the environment under the “volume-based tax” mode. There is a large gap between the grade of raw ore, sales price, and production technology in different regions [2]. The tax method based on quantity cuts the link between price and tax. There is no standardized and unified tax standard in the process of iron ore resource tax, which is likely to reflect the value of the resource itself and the principle of resource scarcity. The production capacity of different iron ore enterprises is uneven. Many small and medium-sized enterprises still have a lot of income and profits in years when the iron ore price is soaring and the resource tax rate is not high. They continue to produce iron concentrate powder, which is a huge hazard to the environment and resources [3]. At the same time, this tax method has no incentive effect on those enterprises that actively invest in their own enterprise innovation and improve the efficiency of iron ore resource utilization. Therefore, China’s iron ore resource tax “based on quantity” has not been able to effectively regulate the iron ore industry. Therefore, it is necessary to explore the establishment of an effective iron ore resource tax collection mechanism, to regulate the relationship between economic development, environmental protection, and resource conservation.

## 2. Literature Review

The formulation and transformation of China’s resource tax policy has gone through five stages: blank period, ad valorem period, reform pilot period, and comprehensive ad valorem period [4]. The function of the iron ore resource tax should be, to solve the negative externalities and promote the sustainable use of resources, rather than to regulate the differential income [5]. As for the importance of resource tax, many studies have constructed CGE models to conclude that economic losses caused by environmental pollution account for 12.86% of GDP, indicating that more than a tenth of China’s GDP is generated at the cost of the consumption of resources and the environment [6]. China’s imposition of an iron ore resource tax is conducive to the rational development and use of resources, and is very important for adjusting the contradictions and conflicts between China’s environment and economy [7]. The collection of iron ore resource tax is a way of environmental supervision, and plays an important role in the improvement of green all-factor energy efficiency [8]. At present, China’s iron ore resource tax is not reasonable. The regulatory role of the iron ore resource tax itself has gradually become weak. The legal system of the iron ore resource tax needs to be changed [9]. The early reform of the resource tax had a certain negative impact on enterprises and consumers in the resource industry, but in the long-term development, the reform of the iron ore resource tax will reduce predatory resource exploitation and improve the efficiency of enterprises in exploiting iron ore resources [10]. Since China’s resource tax and other taxes will affect the comprehensive tax burdens of enterprises, in order to adjust the comprehensive tax burdens of enterprises, and encourage enterprises to save energy and reduce emissions, we should carry out the reform of resource tax ad valorem [11]. In order to give full play to the role of resource tax in saving resources, protecting the environment, and increasing fiscal revenue, China has gradually promoted the pilot work of resource tax from quantity to ad valorem [12].

In terms of the effect of its iron ore resource tax reform, China’s resource tax reform will have a certain impact on enterprises and consumers [13]. At the same time, the reform of China’s iron ore resource tax should adapt to the comprehensive environmental supervision, take the improvement of the market mechanism as the core, adjust and optimize the allocation of resources, and make the price of mineral resources truly reflect their scarcity [14]. The reform of resource tax has increased the comprehensive tax burdens of enterprises in a short time, but in the long run, the comprehensive tax burdens of expert enterprises will gradually decrease with the enterprise tax burdens [15,16]. The reform of resource tax has increased the burden of resource tax, so it urges enterprises to improve the efficiency of resource utilization without changing the production efficiency, thus achieving the purpose of saving resources and protecting the environment [17,18,19].

In view of the future direction of China’s iron ore resource tax reform, the government should encourage the development of innovative technologies for resource utilization through preferential tax policies [20]. Expanding the scope of resource tax collection can improve the green tax system, which is in line with the concept of green development in the new era [21]. China’s iron ore resource tax legislation should take expanding the scope of taxation as the core, and fully reflect the new development concept and the legal spirit of taxation in the tax rate, tax items, tax links, tax basis, and tax preference design [22]. Whether the function of the iron ore resource tax should be to regulate differential income or to solve externalities, clarifying this fundamental problem is the key to get out of the iron ore resource tax dilemma [23]. To reflect the scarcity of iron ore resources and the externalities of mining, the tax rate of iron ore resources should be different according to the reserves of different resources [24]. Other studies have shown that resource tax reform can reduce carbon emissions and increase employment in the medium and long term [25].

Many scholars have discussed the important role of China’s iron ore resource tax and the relationship between the iron ore resource tax and the environmental supervision system, but there is still a gap in the research on the policy effect of China’s iron ore resource tax collection reform. This paper summarizes the policy orientation of China’s iron ore resource tax from “volume-based” to “ad valorem”, and analyzes the impact of policy changes on enterprises’ tax burdens, industrial pollutant emissions, production technology innovation and upgrading, and the number of large and medium-sized industrial enterprises after the reform of the iron ore resource tax. At the same time, this paper demonstrates the environmental and economic effects of the reform of China’s iron ore enterprise resource tax collection mode, and puts forward specific measures to establish an effective iron ore resource tax collection mechanism in China, so as to adjust the relationship between economic development, environmental protection, and resource conservation, and lay the foundation for China to achieve the goal of carbon peak and carbon neutrality, and the reform of the ecological civilization system.

## 3. Methodology and Research Design

### 3.1. Research Hypothesis

China’s resource tax collection method has been changed from “volume-based tax” to “price-based tax”, which has led to the control and improvement of the previous phenomenon of “increasing production without increasing income”. As the former resource tax revenue increase method is based on quotas, many drawbacks gradually began to appear [26]. Because the resource tax is levied on a volume basis, the growth of resource tax revenue can only be attributed to the growth of the amount of iron ore mined. Although the national government has also raised the tax rate, the enterprises have expanded their production scale on the basis of profits for their own economic development, resulting in no significant increase in the tax burden [27]. At the same time, the resource tax income obtained by the local government has not risen significantly, so simply raising the tax rate does not create more tax revenue for the country [28]. On the contrary, because of the profit purpose of enterprises, the mining of mineral resources has been accelerated.

However, the change of the tax method not only increases the tax burden, but also differs from the previous low tax “tax by volume”:

(1) It can optimize the allocation of natural resources and adjust the role of corporate income. Under the condition that the annual output of iron ore enterprises remains unchanged, the increase in the price of iron concentrate powder will greatly increase the incomes and profits of the enterprises. However, if the tax is levied on a volume basis, the relative tax burden will decrease when the profits of the enterprises rise [29]. Therefore, compared with the large profits that iron ore enterprises have received from mining iron ore, they actually pay very little tax. If the tax method is changed to “ad valorem taxation”, the tax intensity will increase as the enterprises’ incomes and profits increase. This will not only make the incomes and profits of iron ore enterprises match the tax, but also strengthen the regulatory role of resource tax on iron ore enterprises.
(2) The actual output of iron ore cannot be calculated, due to the specific collection. Due to the great difference between different enterprise processes, resources, and enterprise systems, it is difficult to directly, objectively, truly and scientifically determine the beneficiation ratio (the ratio to measure the output), which brings considerable difficulty to tax authorities [30]. At the same time, there are many related small and medium-sized enterprises that do not establish accounts, record truthfully, or even issue invoices. It is difficult to monitor tax collection and management. There are a lot of opportunities for “tax evasion”, and the phenomenon of enterprises stealing is also common [31]. The ad valorem tax method can reduce many such phenomena, make the tax system more standardized, reduce the opportunities and space for tax evasion, and to some extent, promote the increase of government revenue. “Ad valorem taxation” is different from “volume-based taxation”. It is based on price. Financial indicators such as sales, gross industrial output value, and gross sales revenue related to price will enter China’s financial statistics system, and the resource tax owed will be more reliable and reasonable [32]. At the same time, it has strengthened the supervision in the process of resource tax collection, standardized the process of resource tax collection, and provided a guarantee for the security and stability of government fiscal revenue.

**Hypothesis 1:**  *The reform of iron ore resource tax can promote the increase of iron ore resource tax revenue.*

**Hypothesis 2:**  *The reform of iron ore resource tax can effectively reduce the emission of industrial pollutants.*

**Hypothesis 3:**  *The reform of iron ore resource tax can effectively improve the production technology of enterprises.*

**Hypothesis 4:**  *The reform of iron ore resource tax will increase the number of large and medium-sized industrial enterprises.*

When collecting the resource tax of iron ore enterprises by “volume-based taxation”, due to the low tax rate, no matter how poor the production technology of the iron ore enterprises and the raw products they use, they can also expand the after-tax profits through large-scale production, resulting in many pollution problems that are difficult to solve, which has a large externality for the environment [33]. The change in the way enterprises levy resource tax can encourage enterprises to abandon the predatory mining and extensive production methods of the past, improve their own production technology and resource utilization rate, improve the mining rate of iron ore and energy utilization rate, and reduce environmental pollution emissions by appropriately increasing the tax burden of enterprises [34].

The “ad valorem” tax collection of resource tax regulates the relationship between enterprise output and tax revenue, and improves the previous situation that in many cases, the higher the enterprise’s output is, the lower the tax intensity of resource tax is, thus forcing enterprises to re-plan their own production technology and production scale, and abandoning their previous behavior of blindly expanding their own production scale. Enterprises can effectively reduce their own operating costs and improve the production utilization of iron ore by improving their own production technology [35]. At the same time, driven by competition in the market, enterprises are forced to change by the compression of their own marginal costs and profit space. For the sake of their own operation and future development prospects, enterprises will consciously increase the importance of innovation investment and improve their own production technology. Therefore, the reform of resource tax revenue mode increases the incentive effect of enterprise innovation [36]. Therefore, this paper believes that the macro resource tax reform can reduce the pollution emissions of iron ore enterprises from two aspects: enterprise production technology and enterprise production scale, which has a positive impact on improving the environment and saving resources [37].

Under the background of “volume-based taxation”, i.e., before the reform of resource tax, there are many small and medium-sized iron ore enterprises whose accounting is not true or even not established, which increases the difficulty of tax supervision of many resource taxes [38]. At the same time, most small and medium-sized iron ore enterprises have a large gap in production technology and raw materials compared with large iron ore enterprises, and their production costs are higher than those of large iron ore enterprises, which will consume more energy and cause more pollution problems [39]. Part of the purpose of the resource tax reform is to eliminate iron ore enterprises with high energy consumption, that are highly polluting, and that cause great environmental damage, so that they will automatically withdraw from the market [40]. The change of the iron ore resource tax from “volume-based tax” to “ad valorem tax” will increase the operating costs of the enterprise itself. For large and medium-sized enterprises, their equipment and production technology are advanced, their production costs are lower than those of other competitors, and their own funds are relatively strong, so they have considerable ability to resist risk [41]. However, for small and medium-sized iron ore enterprises, ad valorem taxation is likely to transform the enterprises from profit-making to loss-making [42]. At the same time, the reduction of small and medium-sized iron ore enterprises will also create opportunities for the establishment of some large and medium-sized iron ore enterprises, and the expansion of their own scale [43]. Therefore, the reform of resource tax will increase the number of large and medium-sized industrial enterprises.

### 3.2. Sample Selection and Data Source

There are large differences in natural resource endowments among provinces in China. Provinces with high resource endowments are greatly affected by resource taxes, while regions with low resource endowments face less resource tax, for them the policy effect of tax reform can be ignored, which makes it possible for this paper to regard resource tax taxation as a “quasi natural experiment” [44]. This paper describes the tax intensity of resource tax by the proportion of resource tax revenue to GDP, reflects the difference of resource endowment among provinces, and divides the experimental group and the control group accordingly [45]. This article takes Shanxi, Inner Mongolia, Qinghai, Shaanxi, and Ningxia, as provinces with high resource tax intensity, as the experimental group, and Sichuan, Henan, Guangxi, Anhui, Jilin, Hubei, Fujian, Tianjin, Jiangsu, Zhejiang, and Guangdong, as provinces with low resource tax intensity, as the control group.

This article uses panel data from 16 provinces in China from 2000 to 2019 to estimate the economic impact of the iron ore resource tax reform. The data used are from the *China Urban Statistical Yearbook*, *China Statistical Yearbook*, *China Science and Technology Statistical Yearbook*, and *China Energy Statistical Yearbook* over the years. In order to reduce heteroscedasticity, this paper logarithmizes some variables.

### 3.3. Variable Definitions

#### 3.3.1. Interpreted Variable

This paper mainly examines the impact of iron ore resource tax reform from four aspects: tax function, environmental governance, production efficiency, and enterprise development. Therefore, this paper chooses resource tax revenue, pollution emissions, the number of large industrial enterprises, and enterprise input–output ratio as the explanatory variables.

(1) Resource tax income. The resource tax revenue is expressed by the sum of the provincial government’s resource tax revenue. The collection method of resource tax is changed from “quantity-based tax collection” to “price-based tax collection”, and the previous tax standard of resource tax is changed from production indicators to more perfect financial statistics indicators, so as to reduce tax evasion, highlight misstatement and financial irregularities, and enhance the stability and security of government financial revenue.
(2) Industrial pollution emissions. One of the purposes of resource tax reform is to reduce environmental pollution and reduce pollution emissions of mining enterprises. In this paper, the sum of emissions of sulfur dioxide, smoke, and nitrogen oxides is selected to measure the improvement effect of resource tax reform on the environment.
(3) Input output ratio. As the main products of iron ore enterprises have been smelted into “pig iron”, “steel”, and “crude steel”, the difference in iron content among the three is very small, so the sum of the three products can be used as the output of iron ore enterprises. In order to measure the progress of an enterprise’s production technology, our input–output ratio can be expressed by the ratio of the output of the iron ore of an industrial enterprise to the industrial enterprise’s investment in innovative R&D [46]. This indicator can measure the output of iron products produced by the enterprise with unit investment, so as to measure the advanced production technology of the enterprise [27]. On the one hand, resource tax reform can promote enterprises to improve production efficiency and production technology, on the other hand, the reform of iron ore resource tax can eliminate iron ore enterprises with high energy consumption, high pollution, and great environmental damage, making them automatically withdraw from the market. For enterprises, for their own future development space, they also need to improve their production technology and increase the requirements for the quality of raw ore materials [47]. Therefore, this paper predicts that the resource tax reform will have a positive effect on the input–output ratio.
(4) Number of large and medium-sized industrial enterprises. Due to the iron ore resource tax reform, the production costs of many small and medium-sized enterprises in the mining industry, that do not meet the requirements of environmental protection and resource protection, suddenly increase, and many enterprises will face financial risks and eventually go bankrupt because of the rising costs. These enterprises, with high energy consumption, high pollution, and great environmental damage will be eliminated from the market due to the reform of resource tax [7]. At the same time, there will also be many medium-sized and large enterprises established and operated on the basis of mergers and acquisitions, which will increase the average size of enterprises in the mining industry. Therefore, this paper predicts that the resource tax reform will have a positive effect on the number of large and medium-sized industrial enterprises.

#### 3.3.2. Core Explanatory Variables

The core explanatory variable ${\mathrm{treat}}_{i}\times \mathrm{period}$ of this paper is the interaction of *dre* and *dt*, and its coefficient represents the impact of the change of the resource tax collection method from “volume-based” to “ad valorem” on provinces and regions with high resource tax intensity.

#### 3.3.3. Control Variable

This paper mainly studies the impact of the change of resource tax collection mode from “volume-based” to “ad valorem” on the government’s resource tax revenue, pollution emissions, and input–output ratio. There are many factors that affect these three policy variables. This paper selects the second industry ratio, the price index of industrial production, the local mining energy consumption (ln_demand), industrial value added (IVA), the number of industrial enterprises (ln_Number), and the number of iron products (ln_output) above the scale of coke consumption, as the control variables that affect these three policy variables. The variable settings and meanings are shown in Table 1.

The proportion of coke consumption (coke pro) is determined by the ratio of coke consumption to the total energy consumption of the mining industry. The main form of energy used by iron ore enterprises is coke, so the change of coke consumption will affect the consumption of energy to a certain extent, thus affecting the three explained variables of resource tax revenue, industrial pollution emissions, and enterprise production technology. The second industry ratio is expressed as the ratio of the output value of the secondary industry to the total output value. The change of the industrial structure can explain the development situation and situation of the industry to a certain extent, thus affecting the resource tax revenue, industrial pollution emissions, and the number of large and medium-sized industrial enterprises [48]. The industrial ex-factory price index will affect the production power and output of industrial enterprises to a certain extent, thus affecting the explained variables in this paper. Local mining energy consumption (ln_demand) and industrial value added (IVA) affect the four explained variables by influencing the output and production costs of iron ore enterprises, to a certain extent. The number of industrial enterprises (ln_Number) will have an impact on the overall output of the mining industry, thus affecting the explained variables. Similar to the influence mechanism of the control variables given above, the output of iron products will also directly affect the government’s resource tax revenue, environmental pollution, enterprise input–output ratio, and enterprise scale, thus affecting the experimental results.

#### 3.3.4. Model Settings

The benchmark regression model in this paper is:

(1) $$Y_{it}=\alpha_{0}+\alpha_{1}treat_{i}+\alpha_{2}period_{t}+\alpha_{3}did_{it}+\alpha_{4}X_{it}+\varepsilon_{it}$$

In Formula (1), i is the province, t is the year, and $Y_{it}$ is the explained variable, which is the resource tax revenue, industrial pollution emissions, enterprise input–output ratio, and the number of large and medium-sized industrial enterprises. $treat_{i}$ is a policy grouping variable. The province considered as the processing group is 1, and the province of the control group is 0. $period_{t}$ is the policy time variable, which is 1 after the policy reform timepoint and 0 before the policy reform timepoint. $did_{it}$ represents the interaction item $treat_{i}\times period_{t}$, which is the product of the policy dummy variable and the time dummy variable, and represents the effect of the treatment group after the implementation of the policy, namely the treatment effect. $X_{it}$ is the control variable. The descriptive statistical results are shown in Table 2 below.

## 4. Analysis of Empirical Results

On the basis of the time fixed effect and the regional fixed effect, we evaluated the policy effect of the iron ore industry resource tax reform from four different dimensions: the government’s resource tax revenue, industrial pollution emissions, input–output ratio, and the number of industrial enterprises. The regression results are shown in Table 3.

Models (1), (3), (5), and (7) are regression results that only consider the time effect and regional effect, without considering control variables; models (2), (4), (6), and (8) are regression results considering control variables, time effects, and regional effects. Comparing the results between the two, we can find that the goodness of fit of the model is significantly improved after adding the control variables.

Model (2) is the regression result of the iron ore resource tax reform on the government’s fiscal revenue. From the perspective of the significance of the regression, the core explanatory variable, DID, is significantly positive at the level of 5%, indicating that the reform of the iron ore industry resource tax can effectively improve the financial revenue. This is because the “ad valorem tax” could optimize the allocation of natural resources, adjust the income of enterprises, and improve the income of enterprises. When the income and profit of enterprises increase, the tax intensity will also increase, which makes the income and profit of iron ore enterprises match the tax, and brings about the increase of financial revenue.

Model (4) is the regression result of iron ore industry resource tax reform on pollution emissions. In terms of the significance of the results, the core explanatory variable DID is not significant, indicating that the reform of the iron ore industry resource tax has no significant impact on the pollution emissions of industrial enterprises. From the perspective of the mechanism, by properly increasing the tax burden of enterprises, enterprises are encouraged to abandon the predatory mining and extensive production methods of the past, and the effect of reducing the emission of environmental pollution is not significant. This may be caused by exogenous factors, which will be further discussed below.

Model (6) is the regression result of iron ore industry resource tax reform on enterprise production technology. From the perspective of the significance of the results, the core explanatory variable DID is significantly positive, at the level of 5%, indicating that the iron ore industry resource tax has a relatively significant improvement effect on the production technology of enterprises. From the perspective of the mechanism, the reform of iron ore resource tax will force the input–output ratio of iron ore enterprises to increase, thus improving the mining rate and energy utilization rate of iron ore, and significantly improving the production technology and resource utilization rate of the enterprises themselves.

Model (8) is the regression result of the industrial resource tax reform of iron ore enterprises on the number of large and medium-sized industrial enterprises. From the significance of the results, the core explanatory variable DID is significantly positive at the level of 5%, indicating that the reform of the iron ore industry resource tax is more beneficial to the development of large and medium-sized industrial enterprises. For large and medium-sized iron ore enterprises, their equipment and production technology are advanced, and their production costs are lower than those of other competitors. However, for small and medium-sized iron ore enterprises, ad valorem taxation is likely to transform the enterprise from profit-making to loss-making, creating opportunities for the establishment of large and medium-sized iron ore enterprises and the expansion of their own scale. Therefore, the reform of resource tax will increase the number of large and medium-sized industrial enterprises.

Through comprehensive comparative analysis of the results in Table 3, we can see that Hypotheses 1, 3, and 4 have been tested and confirmed, but Hypothesis 2 does not meet our expectations, and further research is needed.

The regression results show that the regression direction of Hypothesis 2 (iron ore resource tax reform can effectively reduce industrial pollution emissions) is in line with our expectations, but the results obtained are not significant. After consulting the data, we believe that the policy effect of the newly promulgated Environmental Protection Law of the People’s Republic of China (the most stringent environmental protection law) in 2015, has had a great exogenous impact on the results of this experiment. Therefore, further research will be carried out, taking the amount of environmental pollution as the explanatory variable and the implementation time of the policy of the Environmental Protection Law of the People’s Republic of China as the explanatory variable, to explore whether the emission of industrial pollution in each province has significant changes before and after the implementation of the policy of the Environmental Protection Law of the People’s Republic of China, so as to explore the extent of its impact on the emission of industrial pollution [49]. See Table 4 for the specific regression results.

The above is the regression result of the impact of the implementation of the Environmental Protection Law of the People’s Republic of China on industrial pollution emissions. From the significance of the results, the time of the core explanatory variable is significant at the level of 1%, indicating that it has a very significant impact on industrial pollution emissions. In terms of the direction of action, the core explanatory variable time (post) is negative, indicating that the implementation of the Environmental Protection Law of the People’s Republic of China had an obvious inhibitory effect on industrial pollution emissions. This is in line with our previous speculation, so this paper speculates that hypothesis 2 is correct, but the results are not significant, which is likely due to the external impact of the implementation of the Environmental Protection Law of the People’s Republic of China on industrial pollution emissions [3].

## 5. Robustness TEST

### 5.1. Whether the Selection of Provinces Is Random

The reform of resource tax is not an external event that occurs specifically for experimental purposes. When China changed the tax method from “volume-based” to “ad valorem tax”, it did not specifically customize the tax rate for specific provinces, nor did it clearly announce which provinces would carry out the resource tax reform. At the same time, from the scope of sample selection, the geographical location of the pilot cities is also naturally distributed, and there is no regional correlation, which also provides the experimental basis for the quasi-natural experiment for this study.

### 5.2. Parallel Trend Test

Since the specific time of the reform of the resource tax was 1 July 2016, considering a series of factors, such as that the implementation of the policy only started in the middle of 2016, the specific implementation of the policy may take a certain time, and the iron ore enterprises need a certain adaptation time, this paper adopts 2017, which is one year later, as the full implementation year of the policy [50]. One of the most important and basic prerequisites for the application of DID, is the false premise that as long as the development trends of the experimental group and the control group are consistent before the reform, there can be differences between them, the control group can be considered as the appropriate “experimental control group”.

The parallel trend test methods mainly include the graphic method and parameter method. This paper will test the parallel trend through the parameter method. The parameter method takes the difference of resource tax revenue, pollution emissions, and input–output ratio as the explanatory variable, and the treatment group as the main explanatory variable. The change characteristics of the experimental group and the control group before 2016 are studied. The specific regression results are shown in Table 5:

It can be seen from the results in Table 5 that the regression results of the four model treatment groups are not significant. It can be seen that the resource tax revenue, pollution emissions, and input–output ratio of the provinces in the treatment group and the control group have no significant differences before the resource tax reform, and the parallel trend test is satisfied.

### 5.3. Replace Interpreted Variable

Because the double difference model can solve the problem of causal identification caused by endogenous problems well, this paper controls individuals and time using multiple regression, and adopts a two-way fixed effect model. Therefore, the results of this paper are less disturbed by endogenous problems such as reverse causality and missing variables. However, the results of this paper may be affected by the selection of the year of the reform of the resource tax. Since the specific time for the change of the “specific quantity tax” to “specific price tax” of the iron ore resource tax is 1 July 2016, the year selected in this paper is 2017. In order to better study the effect of the reform of the iron ore resource tax, this paper selects 2016 as the year of policy implementation, on the basis of the above research. On the basis of the revised year, the regression was conducted again, according to the original model, as the robustness test of this paper. When the new year (2016) is used as the year of policy implementation, this paper finds that the empirical results are basically consistent with the previous one (see Table 6), that is, the coefficients in front of the three explained variables of resource tax revenue, enterprise innovation investment ratio, and the number of large and medium-sized industrial enterprises are positive, but the significance of the two explained variables of resource tax revenue and the number of large and medium-sized industrial enterprises has decreased, This may be because the reform of the iron ore resource tax law was implemented on 1 July 2016, and only half a year has passed to test its actual implementation effect, so its actual policy effect may be reduced, but on the whole, the results of the robustness test can further prove the correctness of the year selection.

### 5.4. Placebo Test

The core idea of the placebo test is to re-estimate the original model through the fictitious treatment group or the fictitious policy time. If the coefficient regression result of the interaction item under the fictitious mode is still significant, it means that the original estimation result is likely to be biased, and the change of the explained variable is likely not caused by the policy impact in the model. In this paper, the placebo test was carried out on a fictitious treatment group. The general practice of the placebo test on the treatment group is to select a known group that is not affected by the policy, randomly define it as a “pseudo-experimental group” and a “pseudo-control group”, and then conduct a double difference estimation again. Since the resource tax intensity of the provinces in the control group of the above experiments was originally at a very low level, it can be roughly seen as not affected by the iron ore resource tax reform policy [51]. Therefore, we randomly define a “pseudo-experimental group” and set up interaction items in these provinces, and then use the double difference model to estimate again, focusing on the significance of the coefficients of interaction items. In order to ensure the robustness of the experimental results, two experiments were randomly divided.

Table 7 shows the regression results of the placebo test. Model 2, model 4, model 6, and model 8, respectively, show whether the results under the random grouping are the same as the previous empirical results. It can be seen that the interaction coefficient of the “pseudo experimental group” and the “reform time” dummy variables has not passed the significance test, which indicates that the change trend between the resource tax revenue, industrial pollution emissions, production technology of enterprises, and the number of large and medium-sized industrial enterprises in different provinces may be very close, thus proving that the estimation results of this paper using the double difference model are robust.

## 6. Conclusions

Based on the balanced panel data of 16 provinces in China from 2011 to 2019, this study estimated the impact of iron ore resource tax reform on the government’s resource tax revenue, industrial pollution emissions, enterprise production technology, and the number of large and medium-sized industrial enterprises, using the method of double difference, which better solved the endogenous problems that were difficult to deal with in previous studies. The empirical results show that:

(1) The reform of iron ore resource tax has a relatively significant role in increasing the government’s resource tax revenue, and the resource tax revenue of the treatment group is 58.6% higher than that of the control group on average.
(2) The reform of iron ore resource tax has a restraining effect on industrial pollution emissions, but the actual effect is not significant.
(3) The reform of iron ore resource tax can significantly stimulate enterprises to carry out the transformation of production technology. The input–output ratio of the treatment group is 0.923 higher than that of the control group on average, which shows that the reform of iron ore resource tax can significantly improve the production capacity of enterprises, promote resource conservation, and reduce environmental pollution [52].
(4) The reform of iron ore resource tax can effectively increase the number of large and medium-sized industrial enterprises. The average number of the treatment group is 456.6 higher than that of the control group. This shows that the reform of iron ore resource tax can effectively increase the number of large and medium-sized industrial enterprises, eliminate some small and medium-sized enterprises with high energy consumption, high pollution, and out-dated production technology, and make the iron ore industry more standardized [53]. It can be seen that the change of iron ore resource tax from quantity to ad valorem is indeed conducive to the effective use of China’s resources, and can better transform resources into China’s fiscal revenue. At the same time, the reform of iron ore resource tax can rely on tax and the market to regulate enterprises and eliminate small and medium-sized iron ore enterprises with high costs, high energy consumption, and high pollution [54]. On the one hand, it can promote the innovation of production technology of iron ore enterprises by raising the tax rate, pave the way for realizing the combination of iron ore enterprises’ advantageous resources, and expanding the average scale of iron ore enterprises, while at the same time, it can adjust the production scale of iron ore enterprises to a certain extent, so the purpose of reducing industrial pollution emissions is achieved.

However, after the reform of the tax system, the transition from “tax based on quantity” to “tax based on price” has also brought some new problems to the tax system. Many enterprises can still evade the payment of resources in some ways [55]. These enterprises mainly evade tax in two ways. First, in the daily sales and management of iron ore enterprises, for customers who do not need sales invoices to conduct cash transactions, the sales revenue is not as good as the account or part of the account. For some customers, who do not attach importance to sales invoices, the sales revenue on the bill is reduced in the process of invoicing, to reduce the tax paid. Secondly, in the process of development and utilization of mineral resources, there is a link of dividing ore into usable ore and waste rock and waste ore [56]. Some enterprises divide the available ore into waste rock and waste ore for low-price sales. Such enterprises tend to deliberately exaggerate the amount of waste rock and waste ore in order to reduce the sales revenue on the account, not due to technical mistakes, and avoid paying the resource tax through this wrong division. Due to the complexity of the mining enterprise itself, it is difficult for the tax and financial department to find out whether the enterprise has concealed the tax evasion from the sales unit price and the enterprise’s account [57]. The government should strengthen market supervision and the formulation of laws and regulations, and reduce the occurrence of the above two kinds of tax evasion by strengthening the supervision of the daily transaction flow of iron ore enterprises and the division of ore links [58]. Even in some cases, we can cooperate with other departments, such as the land department and the power department. These departments often have data such as the amount of minerals mined, the grade of minerals, and the electricity consumption for mining mineral resources, from which can be inferred the authenticity and reliability of the enterprise’s book data. When formulating relevant legislation, the government should increase the punishment of iron ore enterprises that conceal their own income, increase the importance of enterprises to social benefits, and let enterprises improve their own business structure and establish modern sustainable development enterprises, while establishing the development concept of integrating economic benefits and social benefits.

## Figures and Tables

**Table 1:** Variable definition and interpretation ^1^.

Variable Name	Code	Definition
Resource tax income	source	Logarithmic value of the government’s resource tax revenue
Pollution emission	ln_emm	Logarithmic value of industrial pollution emissions
Number of large industrial enterprises	ln_Number	The logarithm of the number of large and medium-sized industrial enterprises
Enterprise input–output ratio	ROI	Ratio of output of iron ore enterprises to R&D investment in innovation
Industrial structure (explanatory variable)	second industry ratio	Output value of secondary industry/total output value
Ex-factory price index of industrial production	Price index	Ex-factory price index of industrial production
Energy demand of mining industry	ln_demand	Logarithmic value of mining energy consumption
Industrial output	IVA	Increase in total industrial output value
Proportion of coke consumption in total energy consumption	coke pro	Ratio of coke consumption to total energy consumption
Number of industrial enterprises	ln_number	The logarithm of the number of all industrial enterprises above a certain scale
Iron product output	ln_output	Logarithmic value of total output of iron ore enterprises

^1^ Self-made by the author.

**Table 2:** Data descriptive statistical results ^1^.

Variable Name	Sample Size	Average	Median	Standard Deviation	Minimum	Maximum
ln_source	144.000	3.016	2.856	1.076	−0.315	5.948
ln_emmi	144.000	4.833	4.877	0.684	2.780	5.944
ln_Number	144.000	2629.049	1469.000	2732.178	88.000	10,703.000
second industry ratio	144.000	45.469	45.500	5.439	33.300	62.000
price index	144.000	100.540	99.030	4.943	87.670	119.400
ln_demand	144.000	5.505	5.767	1.495	0.784	8.012
IVA	144.000	8.799	8.979	1.084	6.029	10.575
coke pro	144.000	12.415	3.324	45.102	0.481	449.799
ln_Number	144.000	15,272.639	8835.000	14,996.421	386.000	55,394.000
ln_output	144.000	7695.589	6526.490	6532.945	197.210	33,576.100

^1^ Data sources: China Economic Network, *China Urban Statistical Yearbook, China Statistical Yearbook, China Science and Technology Statistical Yearbook, China Energy Statistical Yearbook*.

**Table 3:** Statistical table of regression results (1) ^1^.

Model	(1)	(2)	(3)	(4)	(5)	(6)	(7)	(8)
Variables	ln_source	ln_source	ln_emmi	ln_emmi	ln_Num	ln_Num	ROI	ROI
DID	0.755 **	0.586 **	−0.0733	−0.0240	315.4 ***	454.6 **	0.864 ***	0.923 **
	(0.283)	(0.270)	(0.120)	(0.132)	(99.30)	(170.4)	(0.288)	(0.423)
Control variable		control		control		control		control
Constant	2.466 ***	15.40 **	5.154 ***	3.648	2638 ***	−812.6	4.272 ***	8.044
	(0.0903)	(6.374)	(0.0362)	(4.175)	(85.82)	(4131)	(0.200)	(14.38)
Time effect	Yes	Yes	Yes	Yes	Yes	Yes	Yes	Yes
Regional effect	Yes	Yes	Yes	Yes	Yes	Yes	Yes	Yes
Observations	144	144	144	144	144	144	144	144
Number of codes	16	16	16	16	16	16	16	16
R-squared		0.678	0.826	0.870	0.347	0.480	0.292	0.399

^1^ Data sources: China Economic Network, *China Urban Statistical Yearbook, China Statistical Yearbook, China Science and Technology Statistical Yearbook, China Energy Statistical Yearbook*. *** *p* < 0.01, ** *p* < 0.05, * *p* < 0.1.

**Table 4:** Statistical table of regression results (2) ^1^.

Variables	ln_emmi
Post	−0.797 ***
	(0.0893)
Constant	5.154 ***
	(0.0357)
Time effect	Yes
Regional effect	Yes
Observations	144
Number of codes	16
R-squared	0.826

^1^ Robust standard error in parentheses: *** *p* < 0.01, ** *p* < 0.05, * *p* < 0.1.

**Table 5:** Parallel trend test results ^1^.

Variables	(1)	(2)	(3)	(4)
	ln_source	ln_emmi	ln_Number	ROI
Treat	7.420	−4.422	−37.36	0.0960
	(4.774)	(21.35)	(32.12)	(0.148)
Constant	0.736	−17.59	28.16	−0.226 *
	(3.376)	(15.09)	(22.71)	(0.104)
Time effect	Yes	Yes	Yes	Yes
Regional effect	Yes	Yes	Yes	Yes
Observations	10	10	10	10
R-squared	0.232	0.005	0.145	0.050

^1^ Robust standard error in parentheses: *** *p* < 0.01, ** *p* < 0.05, * *p* < 0.1.

**Table 6:** Statistical table of regression results (3) ^1^.

Model	(1)	(2)	(3)	(4)	(5)	(6)	(7)	(8)
Variables	ln_source	ln_source	ln_emmi	ln_emmi	ln_Num	ln_Num	ROI	ROI
DID	0.723 **	0.468 *	−0.0734	0.00943	216.8 **	372.7 **	0.779 **	0.739 *
	(0.275)	(0.227)	(0.114)	(0.133)	(98.08)	(150.6)	(0.266)	(0.410)
Control variable		control		control		control		control
Constant	2.466 ***	11.12	5.154 ***	3.630	2638 ***	−4193	4.272 ***	1.299
	(0.0897)	(7.356)	(0.0364)	(4.524)	(85.60)	(4709)	(0.199)	(15.12)
Time effect	Yes	Yes	Yes	Yes	Yes	Yes	Yes	Yes
Regional effect	Yes	Yes	Yes	Yes	Yes	Yes	Yes	Yes
Observations	144	144	144	144	144	144	144	144
Number of codes	16	16	16	16	16	16	16	16
R-squared	0.526	0.666	0.826	0.870	0.328	0.468	0.285	0.385

^1^ Robust standard error in parentheses: *** *p* < 0.01, ** *p* < 0.05, * *p* < 0.1.

**Table 7:** Placebo test results ^1^.

Model	(1)	(2)	(3)	(4)	(5)	(6)	(7)	(8)
Variables	ln_source	ln_source	ln_emmi	ln_emmi	ln_Num	ln_Num	ROI	ROI
DID	0.129	0.0515	0.0459	0.155	270.9	173.3	−0.331	−0.516
	(0.276)	(0.188)	(0.204)	(0.162)	(158.7)	(164.6)	(0.423)	(0.306)
		(0.00186)		(0.000893)		(1.607)		(0.00262)
Control Variable		control		control		control		control
Constant	2.196 ***	2.884	5.166 ***	−12.04 *	3539 ***	−2789	3.718 ***	3.133
	(0.111)	(7.487)	(0.0477)	(6.332)	(121.1)	(13,317)	(0.268)	(34.07)
Time effect	Yes	Yes	Yes	Yes	Yes	Yes	Yes	Yes
Regional effect	Yes	Yes	Yes	Yes	Yes	Yes	Yes	Yes
Observations	99	99	99	99	99	99	99	99
Number of codes	11	11	11	11	11	11	11	11
R-squared	0.317	0.457	0.777	0.888	0.447	0.556	0.363	0.534

^1^ Robust standard error in parentheses: *** *p* < 0.01, ** *p* < 0.05, * *p* < 0.1.

## Data Availability

The data presented in this study are available on request from the first author.
